# Balancing Cationicity and Hydrophobicity in Dermaseptin-A4 Generates a Selective Antimicrobial Peptide with Enhanced Therapeutic Potential

**DOI:** 10.3390/antibiotics15080784

**Published:** 2026-08-14

**Authors:** Weichang Li, Wudi Wang, Boyu Chen, Mingwei Sun, Xiaonan Ma, Lei Wang, Chengbang Ma, Yangyang Jiang, Tao Wang, Chris Shaw, Tianbao Chen, Mei Zhou

**Affiliations:** Natural Drug Discovery Group, School of Pharmacy, Queen’s University Belfast, Belfast BT9 7BL, UKt.chen@qub.ac.uk (T.C.);

**Keywords:** antimicrobial peptides, resistance, cationicity, hydrophobicity, selectivity

## Abstract

Background/Objectives: Antimicrobial peptides (AMPs) have emerged as promising alternatives to conventional antibiotics in response to the escalating global threat of antimicrobial resistance (AMR), owing to their potent antimicrobial activity and low propensity for resistance development. However, their clinical application remains limited by poor selectivity and undesirable toxicity toward mammalian cells. Methods: In this study, the naturally occurring frog-derived AMP Dermaseptin-A4 (A4) was selected as a template for rational design. Guided by the principle that optimising the balance between peptide hydrophobicity and cationicity could improve bacterial membrane targeting while reducing interactions with mammalian membranes, three analogues were designed through the targeted modulation of these physicochemical properties. Results: Among the designed analogues, A4-3 exhibited the best overall biological profile. A4-3 maintained a stable α-helical conformation in membrane-mimicking environments and displayed potent antimicrobial activity against tested Gram-positive and Gram-negative bacteria while exhibiting lower haemolytic and cytotoxic effects than the parent peptide. As a result, A4-3 showed improved selectivity, achieving a selectivity index of up to 34.5. A4-3 rapidly eradicated bacterial cells through a membrane-targeting mechanism, leading to membrane disruption and the loss of cellular integrity, and exhibited a low propensity for resistance development following prolonged exposure. A4-3 also retained its antimicrobial activity under physiologically relevant conditions. Conclusions: Collectively, these findings demonstrate that achieving an optimal balance between peptide hydrophobicity and cationicity is an effective strategy for enhancing antimicrobial selectivity without compromising antibacterial activity, highlighting A4-3 as a promising lead candidate for the development of novel antimicrobial therapeutics against drug-resistant bacterial infections.

## 1. Introduction

Since the advent of antibiotics in the 1940s, bacterial infections have become largely treatable. However, the rapid emergence and global spread of AMR have substantially eroded the effectiveness of conventional antibiotics, creating an urgent need for alternative therapeutic strategies [1,2,3]. Among resistant pathogens, Gram-negative bacteria represent a particularly challenging threat due to their intrinsic and acquired resistance mechanisms [4,5]. The outer membrane serves as an effective permeability barrier that restricts antibiotic entry, while resistance determinants located within the periplasm further limit antimicrobial efficacy [6,7]. Consequently, multidrug-resistant Gram-negative pathogens are increasingly associated with treatment failure and elevated healthcare burdens worldwide [8,9].

AMPs have attracted considerable attention as potential alternatives to conventional antibiotics [10,11]. As evolutionarily conserved components of innate immunity, AMPs typically exert antimicrobial activity through direct interactions with bacterial membranes, resulting in rapid membrane disruption and cell death [12]. Unlike conventional antibiotics that often target specific intracellular pathways, AMPs rely primarily on physicochemical interactions with conserved membrane structures, reducing the likelihood of resistance development through single mutational events [13]. Despite these advantages, the therapeutic application of natural AMPs remains limited by insufficient selectivity and undesirable toxicity toward mammalian cells [14,15]. To address these limitations, extensive efforts have focused on modulating peptide physicochemical properties, particularly cationicity and hydrophobicity, which play central roles in governing antimicrobial activity and membrane selectivity [16]. However, enhancing antimicrobial potency often comes at the expense of increased haemolytic and cytotoxic effects, highlighting the persistent challenge of balancing efficacy and biocompatibility [17,18].

Therefore, a deeper understanding of how charge and hydrophobicity collectively influence peptide performance is essential for the rational design of next-generation antimicrobial peptides. In this work, a naturally occurring frog-derived antimicrobial peptide Dermaseptin-A4 from the Blue-sided leaf frog *Agalychnis annae* was selected as a template for rational design. Three peptide analogues were generated through targeted modifications aimed at decreasing hydrophobicity while increasing net positive charge. The antimicrobial activity, toxicity, and selectivity of the resulting peptides were systematically evaluated against a panel of bacterial pathogens. Furthermore, mechanistic studies, salt-sensitivity assays, and resistance-development assessments were performed to investigate their therapeutic potential.

## 2. Results

### 2.1. Design and Characterisation of A4

**Figure 1:**
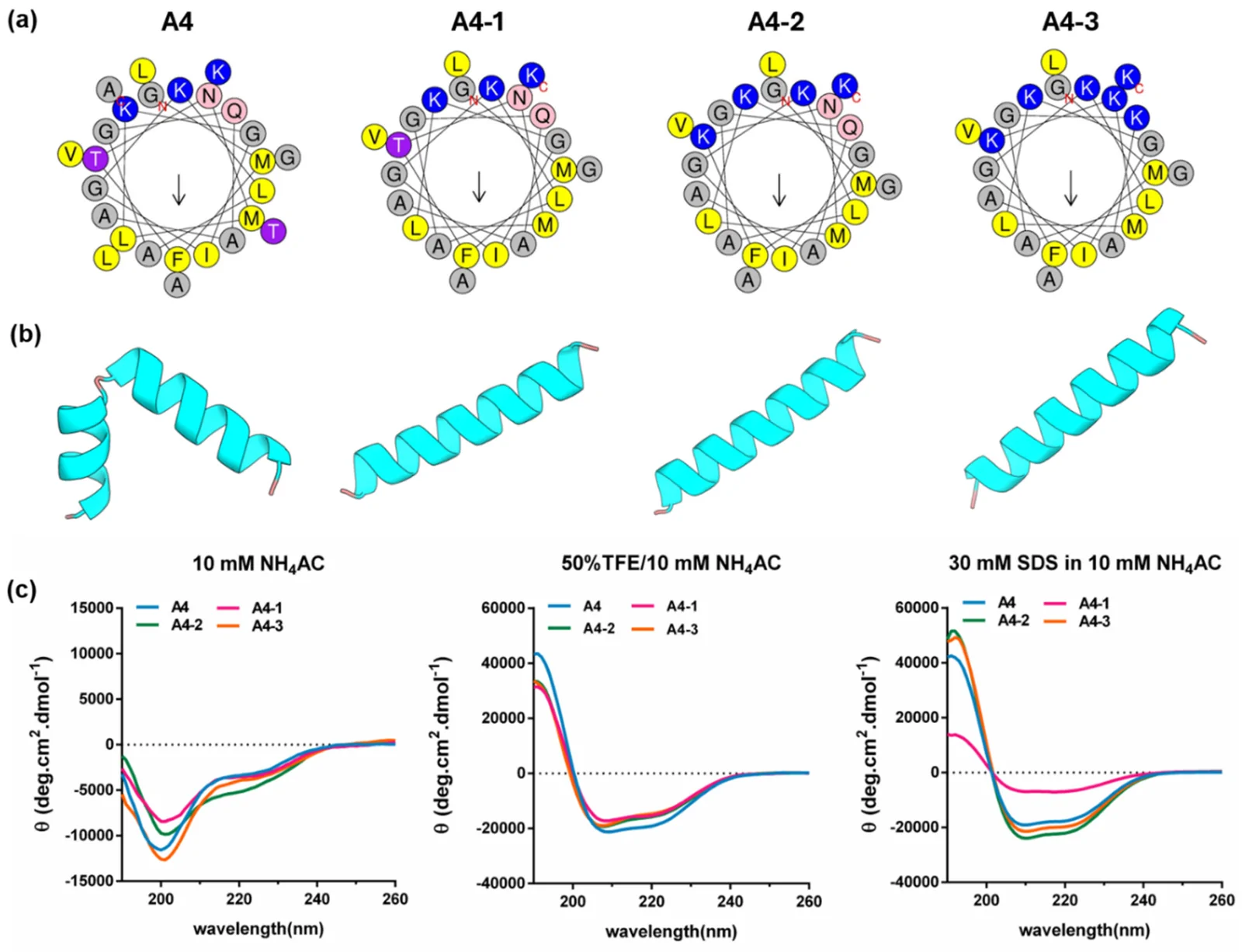
(**a**) Helical wheel projections of A4 and its analogues. Hydrophobic residues are shown in yellow, alanine and glycine residues in grey, threonine residues in purple, lysine residues in blue, and asparagine and glutamine residues in pink. (**b**) Predicted three-dimensional structures of A4 and its analogues. α-Helical regions are shown in cyan, while coil regions are shown in pink. (**c**) CD spectra of A4 and its analogues in NH_4_Ac, TFE, and SDS solutions.

A4, a naturally occurring frog-derived AMP, was selected as the parent template for rational design. Firstly, the three C-terminal residues of A4 were removed to generate A4-1, resulting in a reduction in peptide hydrophobicity from 0.475 to 0.438 while largely preserving the overall amino acid composition (Table 1). Subsequently, positively charged lysine residues were introduced into A4-1 to generate A4-2 and A4-3, leading to a progressive increase in net positive charge. These modifications were designed to alter peptide hydrophobicity and cationicity while maintaining the amphipathic α-helical characteristics of the parent peptide. Helical wheel projections demonstrated that all analogues retained an amphipathic distribution of hydrophobic and hydrophilic residues (Figure 1a). Structural modelling predicted predominantly α-helical conformations for all peptides (Figure 1b). Consistent with these predictions, CD spectroscopy confirmed that A4 and its analogues adopted highly α-helical conformations (>95% helicity) in membrane-mimicking environments, including SDS and TFE, whereas predominantly unordered structures were observed in NH_4_Ac solution (Figure 1c and Table 2).

### 2.2. In Vitro Antimicrobial Activity

A4 exhibited potent antimicrobial activity against both Gram-positive and Gram-negative bacteria, with MIC values ranging from 1 to 16 μM (Table 3). It also retained activity against drug-resistant strains and the clinical MRSA isolate B038 V1S1A, with MIC values ranging from 2 to 16 μM. The removal of the three C-terminal amino acids reduced the antimicrobial activity of A4-1, resulting in a 4- to 8-fold increase in MIC values against the tested Gram-positive bacteria and a 2- to 4-fold increase against the tested Gram-negative strains. This loss of antimicrobial activity was compensated for by increasing the net positive charge in A4-2. Although only limited improvement was observed against the tested Gram-positive bacteria, the antimicrobial activity against Gram-negative bacteria was restored and even slightly enhanced. Further increasing the net positive charge in A4-3 markedly improved its antimicrobial activity, particularly against the tested Gram-negative bacteria, with MIC values ranging from 1 to 2 μM, representing a substantial improvement over the parent peptide.

**Figure 2:**
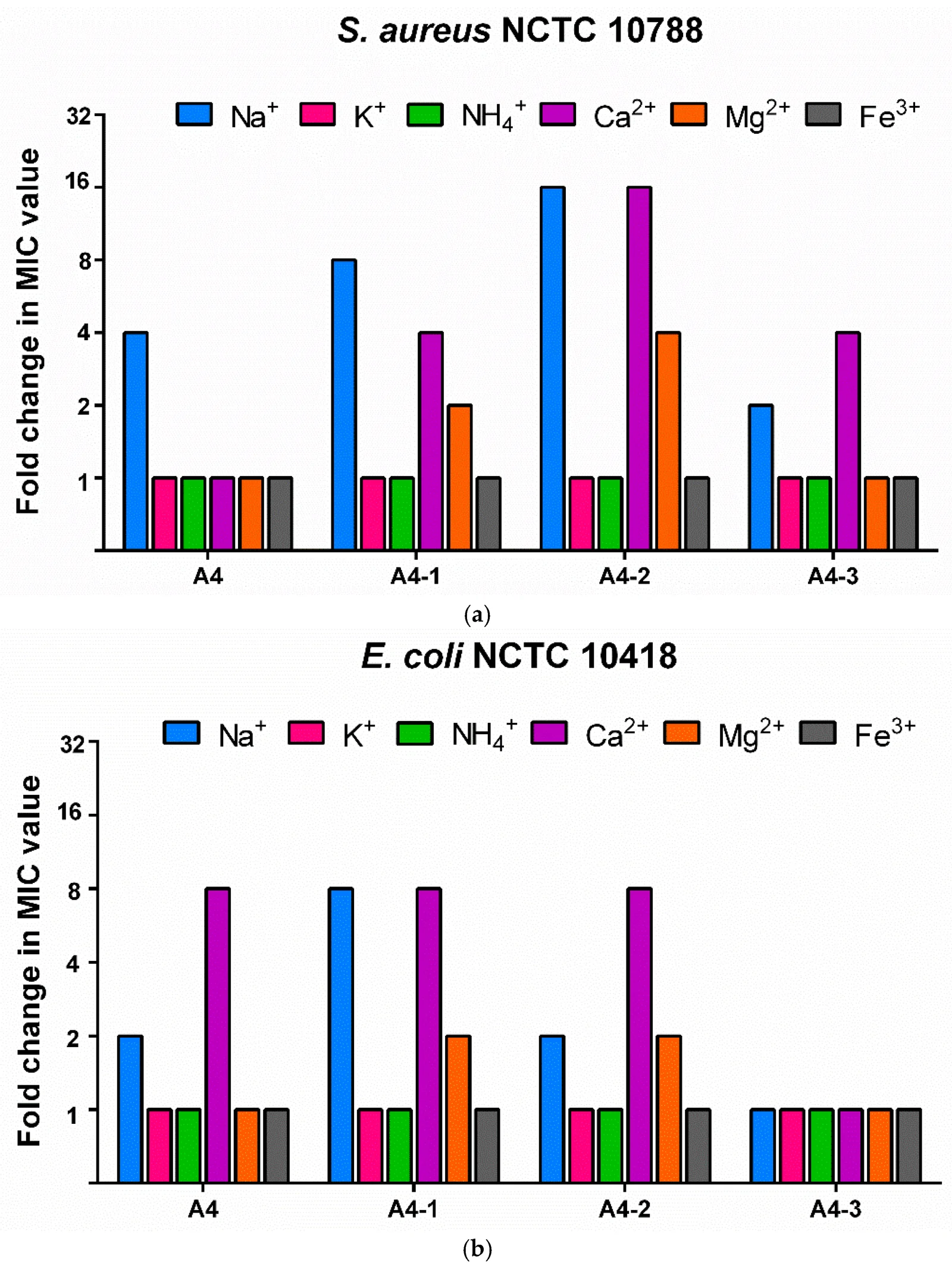
Fold change in MIC values of A4 and its analogues against (**a**) *S. aureus* NCTC 10788 and (**b**) *E. coli* NCTC 10418 in the presence of different salts.

As the antimicrobial activity of many AMPs is often compromised under physiological salt conditions, a salt-sensitivity study was performed to evaluate the activity of A4 and its analogues in the presence of six commonly occurring salts. In this study, *S. aureus* NCTC 10788 and *E. coli* NCTC 10418 were selected as representative Gram-positive and Gram-negative bacterial models, respectively, to provide an initial comparison of salt sensitivity under different ionic conditions. As shown in Figure 2, sodium and calcium ions had the greatest impact on the antimicrobial activity of A4 and its analogues. For A4-1 and A4-2, magnesium ions also increased the MIC values by 2- to 4-fold. In contrast, for A4-3, the presence of salts affected its antimicrobial activity only against S. aureus NCTC 10788, resulting in a 2- to 4-fold increase in MIC values. Against *E. coli* NCTC 10418, these ions had negligible effects on antimicrobial activity, with little or no change in MIC values observed.

### 2.3. Cytotoxicity and Selectivity of A4 and Its Analogues

Horse erythrocytes and human HEK-293 cells were used to evaluate the in vitro cytotoxicity of A4 and its analogues (Table 4). A4 exhibited the highest cytotoxicity among the tested peptides, with an HC_10_ value of 39.1 μM and an IC_50_ value of 19 μM, which were lower than those of the other peptides. The removal of the three C-terminal amino acids not only reduced antimicrobial activity but also weakened interactions with erythrocytes and HEK-293 cells, with an HC_10_ value above 128 μM (the highest tested concentration) and an IC_50_ value of 45.9 μM. Increasing the net positive charge improved the antimicrobial activity of A4-2, accompanied by reduced cytotoxicity toward both erythrocytes and HEK-293 cells. Further increasing the net positive charge enhanced the antimicrobial activity of A4-3. However, cytotoxicity toward mammalian cells also increased, with the HC_10_ decreasing to 76.9 μM and the IC_50_ to 68.1 μM. The selectivity of the peptides was subsequently calculated. Overall, the modifications progressively improved peptide selectivity, and A4-3 exhibited the highest selectivity index (SI) among the tested peptides, with a value of 34.5. In particular, its SI against Gram-negative bacteria (65.8) was higher than that against Gram-positive bacteria. These results indicate that A4-3 is a potent antimicrobial candidate with improved selectivity. Based on these findings, A4-3 was selected for further evaluation.

### 2.4. Time–Kill Kinetics

**Figure 3:**
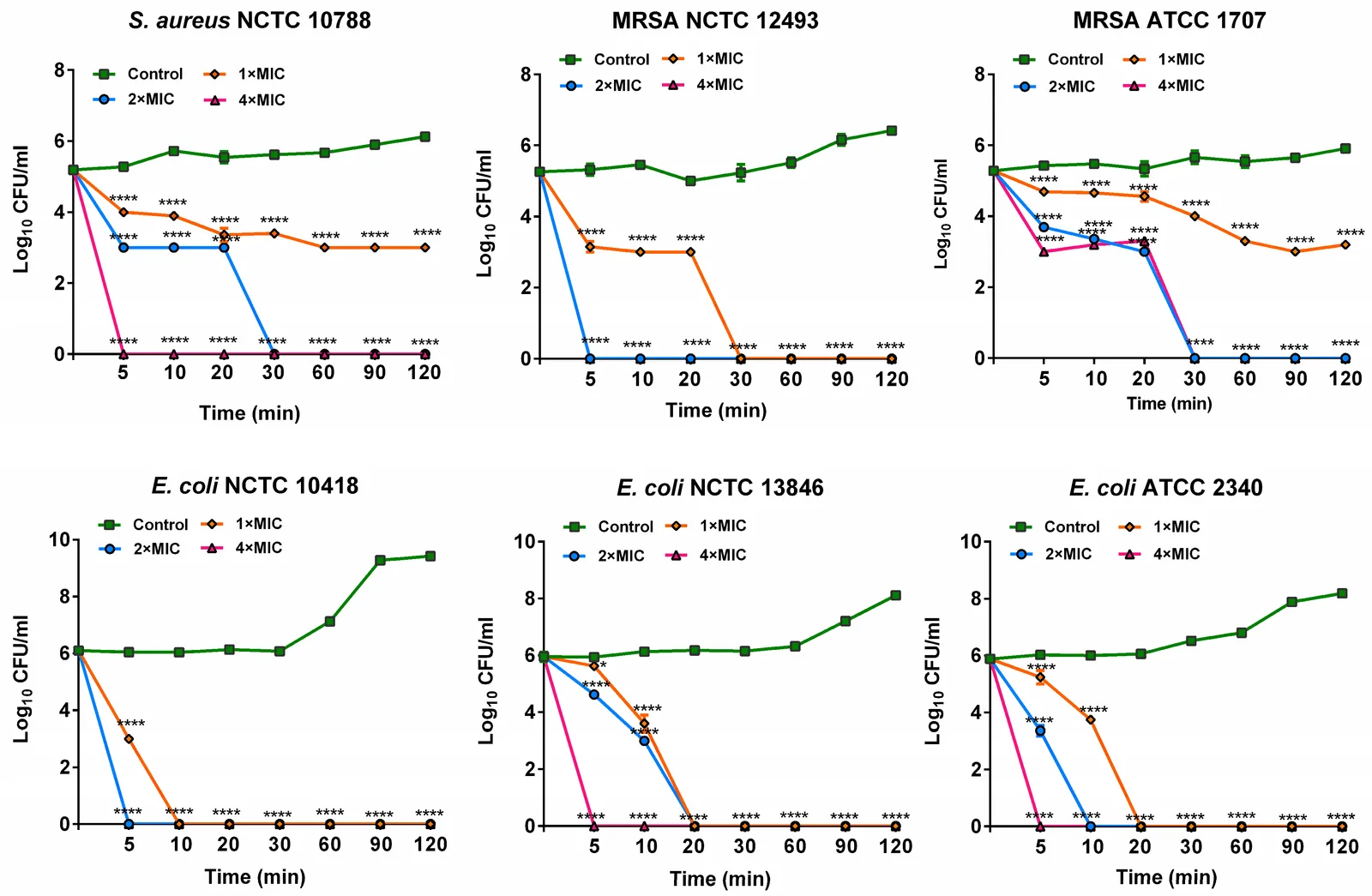
Time–kill kinetic curves of peptide A4-3 against susceptible and resistant strains of *S. aureus* and *E. coli.* Data are presented as mean ± SEM from three independent biological experiments, with each condition tested in triplicate. Statistical analysis was performed using two-way ANOVA followed by multiple comparisons test to compare each treatment group with the untreated control at the corresponding time point. Statistical significance is indicated as * *p* < 0.05 and **** *p* < 0.0001.

Time–kill kinetics of A4-3 were evaluated against susceptible and resistant strains of *S. aureus* and *E. coli* (Figure 3). For the tested *S. aureus* strains, A4-3 at 1× MIC exhibited a bacteriostatic effect against the susceptible strain *S. aureus* NCTC 10788 and the resistant strain MRSA NCTC 1707. At 2× and 4× MIC, A4-3 exhibited bactericidal activity, eliminating bacterial cells within the 120 min observation period. For the tested *E. coli* strains, A4-3 displayed rapid bactericidal activity within the 120 min test period. Even at 1× MIC, A4-3 completely eradicated *E. coli* cells within 20 min, indicating potent and rapid antimicrobial activity.

### 2.5. Antimicrobial Mechanisms Studies

**Figure 4:**
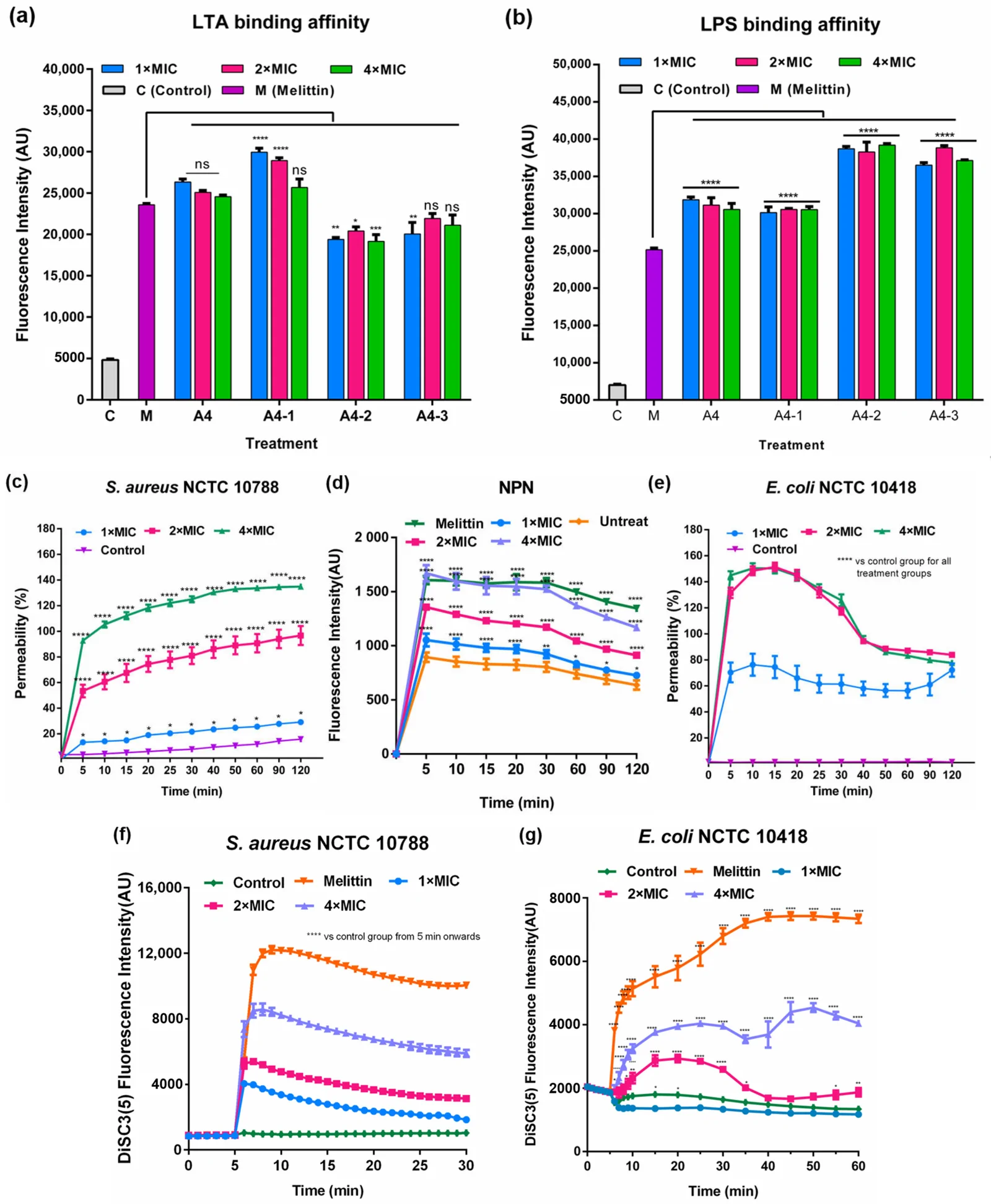
Mechanistic studies of A4-3 against *S. aureus* NCTC 10788 and *E. coli* NCTC 10418. (**a**) LTA-binding affinity of A4-3 compared with the parent peptide and its analogues. (**b**) LPS-binding affinity of A4-3 compared with the parent peptide and its analogues. (**c**) Cytoplasmic membrane permeabilisation of *S. aureus* NCTC 10788. (**d**) Outer membrane permeabilisation of *E. coli* NCTC 10418. (**e**) Cytoplasmic membrane permeabilisation of *E. coli* NCTC 10418. (**f**) Membrane depolarisation of *S. aureus* NCTC 10788. (**g**) Membrane depolarisation of *E. coli* NCTC 10418. Data are presented as mean ± SEM from three independent experiments. Statistical analysis of LTA- and LPS-binding assays was performed using one-way ANOVA followed by Dunnett’s multiple comparisons test, comparing each peptide-treated group with the positive control melittin. Time-dependent membrane permeabilisation and depolarisation assays were analysed using two-way ANOVA followed by multiple comparisons test, comparing each treatment group with the untreated control at each time point. ns, not significant, * *p* < 0.05, ** *p* < 0.01, *** *p* < 0.001, and **** *p* < 0.0001.

*S. aureus* NCTC 10788 and *E. coli* NCTC 10418 were selected as model strains to investigate the mode of action of A4-3. As key components of the bacterial surface in Gram-positive and Gram-negative bacteria, LTA and LPS are generally considered primary binding targets for cationic AMPs due to electrostatic interactions. For LTA, A4 showed comparable binding to melittin, whereas A4-1 exhibited significantly stronger binding and A4-2 significantly weaker binding than melittin (Figure 4a). For A4-3, binding at 1× MIC was significantly lower than that of melittin, while binding at 2 and 4× MIC was comparable to melittin. In contrast, all peptide treatments exhibited significantly stronger binding to LPS than melittin at the corresponding concentrations (Figure 4b). These results indicate that there is a stronger interaction of the tested peptides with LPS than with LTA, with A4-3 showing a potent LPS-binding capacity. This interaction with LPS is consistent with the enhanced antibacterial activity of A4-3 against Gram-negative bacteria.

Subsequently, cytoplasmic membrane permeabilisation of *S. aureus* NCTC 10718 was evaluated using the SYTOX Green assay. As shown in Figure 4c, A4-3 induced concentration-dependent membrane permeabilisation. In the first 5 min, fluorescence increased rapidly and significantly, indicating fast membrane disruption. By the end of the 120 min observation period, membrane permeabilisation reached approximately 20% at 1× MIC, approximately 100% at 2× MIC, and approximately 140% at 4× MIC. For *E. coli* NCTC 10418, the NPN assay was first performed to evaluate outer membrane permeability. As shown in Figure 4d, A4-3 induced a clear concentration-dependent increase in outer membrane permeability. At 4× MIC, the fluorescence signal induced by A4-3 was comparable to that of melittin, indicating similar membrane-disrupting ability. The effect of A4-3 on *E. coli* cytoplasmic membrane permeabilisation was then evaluated. At the initial 5 min, all tested concentrations caused a rapid and significant increase in membrane permeability, reaching a peak within the observation period (Figure 4e).

**Figure 5:**
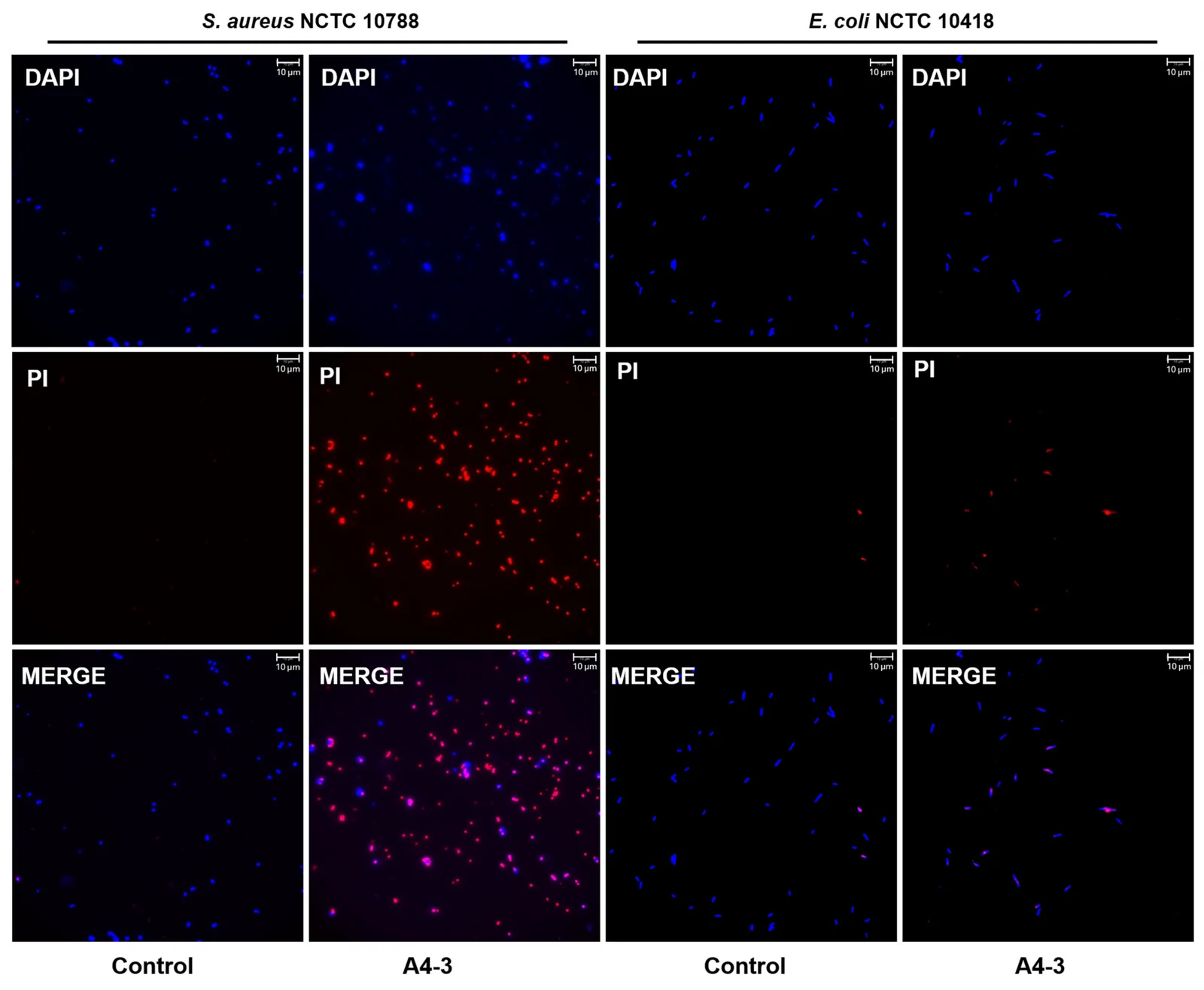
Fluorescence microscopy images of *S. aureus* NCTC 10788 and *E. coli* NCTC 10418 in the absence and presence of A4-3. The scale bar is 10 µm.

Since cytoplasmic membrane damage is typically accompanied by the dissipation of membrane potential, the effect of A4-3 on membrane depolarisation was also assessed. As shown in Figure 4f,g, A4-3 induced a concentration-dependent change in membrane potential. In *S. aureus* NCTC 10788, fluorescence intensity increased rapidly within 5 min and reached a peak shortly thereafter, followed by a gradual decrease, possibly due to dye quenching. In *E. coli* NCTC 10418, fluorescence reached a maximum more slowly, at approximately 40 min. However, all tested concentrations induced lower fluorescence intensity compared with melittin. To further validate the membrane-disrupting effects of A4-3 on both strains, a DAPI/PI staining assay was performed. As shown in Figure 5, compared with the control group, A4-3 treatment induced a marked increase in PI fluorescence intensity in both *S. aureus* NCTC 10788 and *E. coli* NCTC 10418, indicating membrane disruption. Collectively, these results support a membrane-disruption mode of action in which A4-3 initially binds to LTA or LPS on the bacterial surface, followed by rapid membrane permeabilisation and dissipation of membrane potential. These events ultimately lead to membrane damage and bacterial cell death.

### 2.6. Resistance Development Evaluations to A4-3

**Figure 6:**
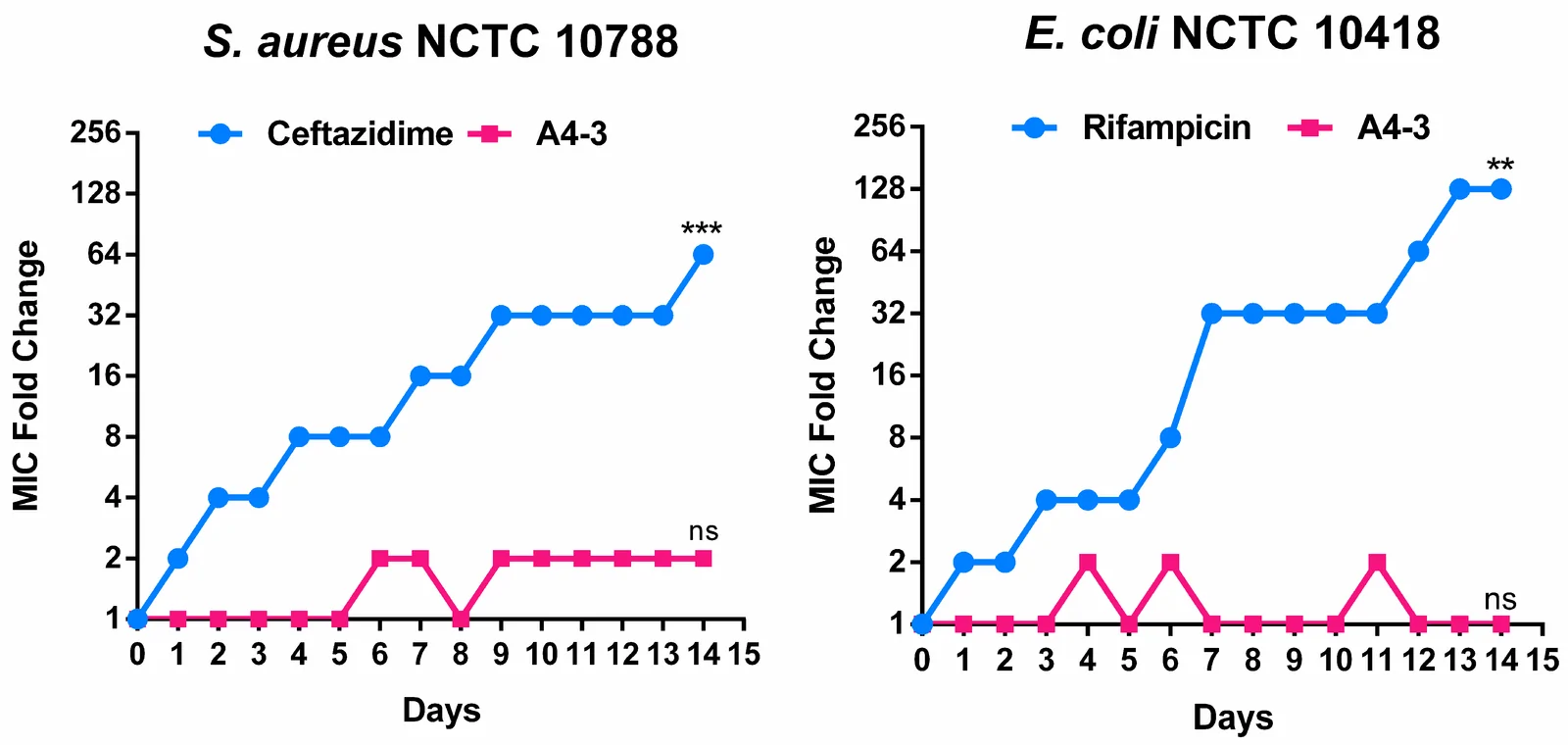
Resistance development of *S. aureus* NCTC 10788 and *E. coli* NCTC 10418 during serial passaging in the presence of ceftazidime/rifampicin and A4-3. Bacterial populations were serially passaged for 14 consecutive cycles in the presence of sub-inhibitory concentrations of A4-3 or antibiotic controls (ceftazidime for *S. aureus* NCTC 10788 and rifampicin for *E. coli* NCTC 10418). MIC fold changes were calculated relative to the initial MIC value (passage 0). Values represent the mean ± SEM from three independent experiments. Statistical significance was determined by comparison with the initial MIC value. ns, not significant, ** *p* < 0.01, and *** *p* < 0.001.

Given that A4-3 exhibited the most promising antimicrobial profile among the designed analogues, it was selected as the representative peptide for resistance evolution studies alongside conventional antibiotic control. The propensity of A4-3 to induce resistance was evaluated using *S. aureus* NCTC 10788 and *E. coli* NCTC 10418 as model organisms. Ceftazidime and rifampicin were included as antibiotic controls, respectively (Figure 6). Following 14 sequential passages, both bacterial strains developed resistance to ceftazidime or rifampicin, as reflected by the progressive increase in MIC values. In contrast, both bacteria remained susceptible to A4-3 throughout the experiment. After 14 passages, the MIC values of A4-3 remained unchanged compared with those recorded on the first day, whereas significant increase in MIC values were observed in both antibiotic control groups compared with the initial MIC values. These findings indicate that A4-3 exhibited a lower propensity to induce resistance than the antibiotics used in the control groups.

## 3. Discussion

Although AMPs are considered among the most promising candidates for next-generation antimicrobial agents, their therapeutic application is often limited by poor selectivity and undesirable toxicity toward mammalian cells [19,20]. Considerable efforts have therefore been devoted to improving AMP selectivity through the modulation of key physicochemical parameters, including net charge, hydrophobicity, amphiphilicity, and secondary structure [21,22,23]. In this work, the naturally occurring frog-derived AMP Dermaseptin-A4 (A4) was selected as a template for rational optimisation. Three analogues were generated through the targeted modulation of hydrophobicity and net positive charge.

The parent peptide A4 exhibited potent antimicrobial activity against both Gram-positive and Gram-negative bacteria, including susceptible and resistant strains. However, its relatively high cytotoxicity resulted in a low selectivity index (SI = 9.8). Previous studies have suggested that the antimicrobial activity of AMPs is governed by a combination of physicochemical properties, including net charge, hydrophobicity, amphiphilicity, and secondary structure [24,25]. Consistent with other members of the Dermaseptin family, A4 adopted a predominantly α-helical conformation in hydrophobic environments. Based on these characteristics, the three C-terminal residues were first removed, reducing hydrophobicity from 0.475 to 0.438. Although this modification had little effect on α-helical formation, it resulted in reduced antimicrobial activity, particularly against the tested Gram-positive bacteria. This observation is consistent with previous reports suggesting that antimicrobial activity against Gram-positive bacteria is generally more sensitive to changes in hydrophobicity [26,27]. In contrast, increasing the net positive charge in A4-2 and A4-3 primarily enhanced activity against Gram-negative bacteria, indicating that cationicity plays a more important role in governing activity against these organisms.

The mechanistic studies provide a possible explanation for these observations. In the LPS-binding assay, the charge-enhanced analogues A4-2 and A4-3 exhibited stronger binding affinity than A4 and A4-1. As LPS contains multiple phosphate and pyrophosphate groups, it possesses a highly negatively charged surface [28,29]. Consequently, peptides with higher net positive charges are more likely to establish stronger electrostatic interactions with Gram-negative bacterial membranes during the initial binding stage. Together with the rapid outer membrane permeabilisation observed in the NPN assay, this enhanced interaction may facilitate subsequent disruption of the cytoplasmic membrane, thereby improving antibacterial activity. In contrast, increasing cationicity did not provide a comparable advantage in interactions with LTA. Although LTA also carries a negative charge, peptide interaction with Gram-positive bacteria appears to rely not only on electrostatic attraction but also on hydrophobic interactions. Following initial surface binding, peptides must penetrate the thick peptidoglycan layer before reaching the cytoplasmic membrane. Under these circumstances, hydrophobicity may play a more important role in membrane insertion and disruption [30]. This may explain why reducing hydrophobicity consistently weakened antimicrobial activity against Gram-positive bacteria. Dermaseptin peptides typically possess moderate positive charges, pronounced amphipathic α-helical structures, and substantial hydrophobic surfaces that contribute to membrane insertion. Therefore, hydrophobicity is likely to be a key determinant of their biological activity. The marked reduction in antimicrobial activity observed following only a modest decrease in hydrophobicity further supports the importance of hydrophobic interactions in the mechanism of Dermaseptin-derived peptides.

The modification results in this work suggest that balancing hydrophobicity and cationicity is an effective strategy for improving the selectivity of antimicrobial peptides. While reducing hydrophobicity may compromise antimicrobial activity to some extent, it can also markedly decrease undesirable toxicity toward mammalian cells, thereby improving overall selectivity. Importantly, the loss of activity can be partially or even fully compensated through the rational enhancement of peptide cationicity. These findings highlight the delicate interplay between hydrophobicity and charge, indicating that an optimal balance between these parameters may be critical for achieving favourable antimicrobial performance and selectivity. Recent studies have further highlighted the importance of balancing cationic interactions and hydrophobicity in the development of selective antimicrobial agents. For example, tertiary alkylamine-functionalised polyaspartamides have been reported to achieve potent antibacterial activity and high selectivity through the optimisation of cationic structures and hydrophobic alkyl chains, demonstrating the broader applicability of physicochemical tuning beyond peptide-based systems [31]. In addition, recent advances in understanding membrane-active antimicrobial polypeptides have emphasised the critical role of peptide structure, membrane interaction, and assembly behaviour in determining antimicrobial activity and selectivity [32]. Consistent with these findings, the present study demonstrates that rational modulation of cationicity and hydrophobicity within a peptide framework can effectively improve antimicrobial performance while maintaining favourable selectivity. Nevertheless, this study represents only an initial step in the development of Dermaseptin-derived antimicrobial agents. Additional challenges, including peptide stability, pharmacokinetic behaviour, and efficacy in more complex in vivo systems, remain to be addressed before clinical translation can be considered. As many AMPs, the peptides described in this work are likely to be susceptible to degradation by serum proteases, which may reduce their stability, bioavailability, and overall therapeutic exposure. In addition, their pharmacokinetic properties, including circulation half-life, tissue distribution, and clearance, have not yet been characterised. Future optimisation aimed at improving these properties may alter the balance achieved in the present study and require further refinement of peptide design. Furthermore, formulation strategies may be necessary to enhance their clinical applicability by improving peptide stability, prolonging systemic exposure, and facilitating efficient delivery to sites of infection.

From a therapeutic perspective, the potential application of A4-3 will also depend on the route of administration and the specific clinical context. In addition, the scalability and cost-effectiveness of peptide production remain important considerations for future clinical development. Given its enhanced activity against the tested bacterial strains, particularly Gram-negative bacteria, together with its favourable selectivity, A4-3 may represent a promising candidate for localised applications against Gram-negative infections, where direct delivery could maximise antimicrobial activity while reducing potential systemic toxicity. Although systemic administration remains an important long-term goal, further evaluation of pharmacokinetic properties, formulation approaches, and in vivo efficacy will be required to determine its feasibility for clinical use. In parallel with these translational considerations, artificial intelligence (AI)-assisted approaches provide new opportunities to accelerate AMP discovery and development. The manual, stepwise peptide modifications used in this study enabled the mechanistic optimisation of cationicity and hydrophobicity but are inherently limited by relatively low throughput and restricted exploration of sequence space. In contrast, AI-driven platforms can rapidly analyse large peptide libraries and predict favourable combinations of physicochemical properties, including charge, hydrophobicity, stability and toxicity, thereby facilitating the identification and development of promising antimicrobial candidates. Recent studies have manifested AI-assisted approaches in accelerating the discovery of anti-*staphylococcal* antimicrobial agents [33,34]. Combining AI-based predictive models with experimentally validated structure–activity relationships may therefore provide a powerful strategy for guiding the future development of AMP analogues with improved therapeutic properties.

Despite these challenges, the rational design strategy employed here successfully generated the analogue A4-3, which exhibited potent broad-spectrum antimicrobial activity, improved selectivity (SI = 34.5), and a low propensity for resistance development. Mechanistic studies demonstrated that A4-3 acts through a membrane-targeting mode of action involving initial electrostatic interactions with the bacterial surface, followed by membrane permeabilisation, membrane depolarisation, and the disruption of cellular integrity, ultimately leading to bacterial cell death. Collectively, these findings identify A4-3 as a promising lead peptide and provide useful insights for the future design of selective AMPs.

## 4. Materials and Methods

### 4.1. Peptide Synthesis and Purification

The peptides used in this study were commercially synthesised and purchased from Motif Biotechnology Co., Ltd. (Motif Biotech, Suzhou, China). The purity of all peptides was >95%, as confirmed by reverse-phase high-performance liquid chromatography (RP-HPLC), and their molecular weights were verified by mass spectrometry.

### 4.2. Circular Dichroism Spectrum Analysis

Circular dichroism (CD) spectroscopy was performed to analyse peptide secondary structure. Peptides (50 μM) were prepared in 10 mM NH_4_Ac, 50% TFE/10 mM NH_4_Ac, or 30 mM SDS in 10 mM NH_4_Ac. NH_4_Ac solutions were prepared by dissolving ammonium acetate in ddH_2_O and adjusting concentrations accordingly. CD spectra were recorded on a J-810 spectropolarimeter (JASCO, Essex, UK) from 190 to 260 nm at 1 nm intervals. Secondary structure contents (α-helix, β-sheet, and random coil) were estimated using the K2D3 web server (http://cbdm-01.zdv.uni-mainz.de/~andrade/k2d3/, accessed on 24 July 2026) [35].

### 4.3. Antimicrobial Assay

Minimal inhibitory concentration (MIC) and minimal bactericidal concentration (MBC) were determined by broth microdilution [36]. Bacteria were grown to log phase in MHB and adjusted to standard OD values. Peptides were serially diluted and incubated with microbial suspensions (5 × 10^5^ CFU/mL) in 96-well plates overnight at 37 °C. MIC was defined as the lowest concentration with no visible growth, and MBC was confirmed by plating on MHA. All assays were performed in triplicate. Salt resistance was assessed using MHB supplemented with various salts (e.g., NaCl, KCl, NH_4_Cl, CaCl_2_, MgCl_2_, FeCl_3_) under the same conditions.

### 4.4. Haemolysis Assay

Fresh defibrinated horse blood (E&O Laboratories Ltd., Falkirk, UK) was used as a mammalian erythrocyte model [36]. Red blood cells were isolated by repeated centrifugation (900× *g*, 5–10 min) and washed with PBS until the supernatant was clear, followed by resuspension to 4% (*v*/*v*). Peptides were prepared in DMSO and serially diluted in PBS. RBC suspensions were incubated with peptides in 2 mL tubes at 37 °C for 2 h. Triton X-100 (1%) and PBS were used as positive and negative controls, respectively. After incubation, samples were centrifuged (900× *g*, 10 min), and supernatants were transferred to a 96-well plate. Absorbance was measured at 570 nm using a Synergy HT microplate reader (Bio-Tek, Winooski, VT, USA).

### 4.5. MTT Assay

Human embryonic kidney cells (HEK-293, Caltag Medsystems, Buckingham, UK) were used to evaluate the cytotoxicity of the peptides [27]. Briefly, cells were cultured in MEM (Gibco, Renfrewshire, UK) supplemented with 10% fetal bovine serum (FBS) (Gibco, Renfrewshire, UK) and 1% penicillin–streptomycin (PS) (Gibco, Renfrewshire, UK) and seeded into 96-well plates for 24 h to allow cell attachment. The following day, the cells were treated with two-fold serial dilutions of the peptides at concentrations ranging from 1.6 to 100 μM. After 24 h of incubation, MTT solution (5 mg/mL) was added, and the cells were further incubated for 4 h at 37 °C to allow the formation of formazan crystals. The medium was then removed, and the formazan crystals were dissolved in DMSO. Absorbance was measured at 570 nm using a microplate reader.

### 4.6. Time–Killing Kinetics Assay

A bacterial suspension was prepared at the required density as described in the antimicrobial assay section. Based on previously determined MIC values, peptide solutions were prepared at 100×, 200×, and 400× MIC in DMSO. Bacteria were incubated with peptides in 96-well plates, resulting in final concentrations of 1×, 2×, and 4× MIC. Growth controls without peptide were included. At defined time points (0–120 min), aliquots were serially diluted in PBS and plated onto MHA agar. Colonies were counted after overnight incubation at 37 °C, and bacterial survival was calculated as CFU/mL = (colony number × dilution factor)/inoculation volume. Time–kill curves were generated based on CFU values over time.

### 4.7. Lipoteichoic Acid & Lipopolysaccharide Binding Assay

Lipoteichoic acid (LTA, Sigma-Aldrich, Dorset, UK) or Lipopolysaccharide (LPS, Sigma-Aldrich, Dorset, UK) was mixed with BC dye (ThermoFisher, Waltham, MA, USA) in 1 M Tris-HCl buffer (pH 7.2) to achieve final concentrations of 50 μg/mL and 5 μg/mL, respectively, followed by incubation at room temperature for 4 h [27,37,38]. Peptide stock solutions were prepared in DMSO and serially diluted in Tris-HCl buffer. Peptides were tested at final concentrations corresponding to 1×, 2×, and 4× MIC. Melittin and Tris-HCl buffer served as positive and negative controls, respectively. All samples were assayed in triplicate in black 96-well plates. Following addition of the LPS–BC or LTA–BC complexes, fluorescence was recorded using a Synergy HT Microplate Reader (Ex 590 nm & Em 645 nm) (Bio-Tek, Winooski, VT, USA).

### 4.8. NPN Assay

Bacteria stored at −20 °C (glycerol or bead stocks) were thawed and cultured overnight in MHB at 37 °C, 200 rpm. On the following day, cultures were subcultured in fresh MHB and grown for 2 h, then washed and resuspended in a background buffer (5 mM HEPES, 5 mM glucose, pH 7.2) to OD600 0.48–0.52. Based on previously determined MIC values, peptides were prepared in DMSO at 100×, 200×, and 400× MIC. Outer membrane permeabilisation was assessed using NPN (80 μM final) in black 96-well plates. Melittin and buffer were used as positive and negative controls, respectively. Bacterial suspensions and peptides were incubated at final concentrations of 1×, 2×, and 4× MIC. Fluorescence was recorded at 360/460 nm every 3 min for 1 h using a Synergy HT microplate reader at 37 °C. Permeabilisation kinetics were plotted based on fluorescence changes over time.

### 4.9. SYTOX Green Assay

Bacteria stored at −20 °C (glycerol or beads) were inoculated into TSB (or NB for MRSA) and cultured overnight at 37 °C, 200 rpm. A salt-containing dilution medium (PBS with 5% culture medium and 0.85% NaCl) was prepared in advance. Overnight cultures were subcultured for 2.5 h, then centrifuged (2000× *g*, 4 °C, 15 min), washed three times, and resuspended in salt-containing medium to the required OD590 (0.68–0.72 for Gram-positive or 0.65–0.70 for Gram-negative). Peptides were prepared in DMSO and diluted to 100–400× MIC. SYTOX Green (50 μM, Life Technologies, Renfrew, UK) was used for membrane permeability assays in black 96-well plates (triplicate). Melittin and medium served as controls. After adding peptides (1 μL) and bacterial suspension (50 μL), fluorescence (Ex/Em 485/582 nm) was recorded every 5 min for 2 h.

### 4.10. Membrane Depolarisation Assay

Bacteria stored at −20 °C (glycerol or bead stocks) were thawed and cultured overnight in MHB at 37 °C, 200 rpm. A background buffer (5 mM HEPES, 20 mM glucose, 0.1 M KCl, pH 7.2) was prepared in advance. On the following day, cultures were subcultured in fresh MHB and grown for 2 h, then washed by repeated centrifugation and resuspended in background buffer to OD600 0.38–0.42. Cells were incubated with DiSC3(5) (2 μM) for 45 min at room temperature to allow dye equilibration. Based on previously determined MIC values, peptides were prepared in DMSO and diluted in background buffer to 10×, 20×, and 40× MIC. For the assay, peptide solutions (10 μL) were added to bacteria–dye suspensions (90 μL) in black 96-well plates, resulting in final concentrations of 1×, 2×, and 4× MIC. Melittin and buffer were used as positive and negative controls, respectively. Fluorescence was recorded at excitation/emission 590/645 nm every 1 min for 60 min using a microplate reader, and time-resolved changes were analysed by integrating pre- and post-treatment signals.

### 4.11. DAPI/PI Staining Assay

Membrane integrity was assessed using DAPI (4′,6-Diamidino-2-pheny lindole dihydrochloride, Sigma-Aldrich, Dorset, UK) and propidium iodide (PI, Sigma-Aldrich, Dorset, UK) staining. Log-phase *S. aureus* NCTC 10788 and *E. coli* NCTC 10418 cells were harvested, washed twice with PBS, and incubated with peptides at 1×, 2×, and 4× MIC for 1 h at 37 °C. Following treatment, cells were stained sequentially with PI (5 μg/mL) and DAPI (10 μg/mL) at 4 °C in the dark, with washing steps performed between staining procedures to remove unbound dye. Fluorescence images were acquired using a Leica DMi8 fluorescence microscope (Leica, Nussloch, Germany) equipped with a 100× oil-immersion objective.

### 4.12. Resistance Induction Assay

*S. aureus* NCTC10788 and *E. coli* NCTC 10418 were used as model organisms to evaluate the potential for resistance development in the presence of peptide A-4, with appropriate antibiotics included as controls [39]. Bacteria were first cultured in medium containing 1/2 MIC of the peptide or antibiotics for 24 h. The cultures were then transferred into fresh mediums containing the same sub-MICs. This serial passage process was continued for 14 consecutive cycles. The MIC values were determined after each cycle to monitor changes in susceptibility over time.

### 4.13. Statistical Analysis

All experiments were performed in at least three independent replicates. Data were analysed using GraphPad Prism 10 (GraphPad, San Diego, CA, USA). Results are presented as mean ± SEM. Statistical significance was determined using appropriate tests in Prism. Statistical significance was determined using one-way or two-way analysis of variance (ANOVA), as appropriate. For comparisons between multiple groups and a single control group, one-way ANOVA followed by Dunnett’s multiple comparisons test was used. For time-dependent experiments, two-way ANOVA followed by multiple comparisons test was applied to compare each treatment group with the corresponding control at individual time points. ns, not significant; * *p* < 0.05, ** *p* < 0.01, *** *p* < 0.001, and **** *p* < 0.0001.

## 5. Conclusions

In summary, the naturally occurring AMP Dermaseptin-A4 was successfully optimised through the modulation of hydrophobicity and cationicity. Among the designed analogues, A4-3 exhibited the most favourable balance between antimicrobial activity and biocompatibility, displaying potent broad-spectrum activity against both susceptible and resistant bacteria while achieving markedly improved selectivity. Mechanistic studies demonstrated that A4-3 exerts its antibacterial activity through a membrane-targeting mode of action involving membrane permeabilisation and depolarisation, ultimately leading to bacterial cell death. Furthermore, A4-3 showed a low propensity to induce resistance. These findings demonstrate that balancing hydrophobicity and cationicity is an effective strategy for improving the selectivity of Dermaseptin-derived AMPs. This work highlights the distinct contributions of hydrophobicity and cationicity to antimicrobial activity against Gram-positive and Gram-negative bacteria and provides useful insights for the rational design of next-generation selective AMPs.

## Figures and Tables

**Table 1:** Sequences and physiochemical properties of A4 and its analogues.

Peptide	Sequence	Net Charge	Hydrophobicity	Hydrophobic Moment
DRS-A4	GMFTNMLKGIGKLAGQAALGAVKTLA-NH2	4	0.475	0.355
A4-1	GMFTNMLKGIGKLAGQAALGAVK-NH2	4	0.438	0.353
A4-2	GMFKNMLKGIGKLAGQAALGAVK-NH2	5	0.384	0.374
A4-3	GMFKKMLKGIGKLAGKAALGAVK-NH2	7	0.333	0.408

**Table 2:** α-Helical contents (%) of A4 and its analogues in NH_4_Ac, TFE and SDS solutions.

Peptide	NH_4_Ac	TFE	SDS
A4	20.5	95.2	95.3
A4-1	36.3	95.2	95.3
A4-2	67.1	95.2	95.3
A4-3	21.3	95.4	95.3

**Table 3:** MIC and MBC values (μM) of A4 and its analogues.

Microorganisms	MIC/MBC			
	A4	A4-1	A4-2	A4-3
*S. aureus* NCTC 10788	1/1	4/4	2/2	1/1
MRSA NCTC 12493	2/2	16/16	16/16	4/8
MRSA ATCC 1707	4/4	16/16	16/32	4/4
MRSA B038 V1S1 A	2/2	16/32	16/32	4/8
*E. faecalis* NCTC 12697	8/8	32/64	32/64	16/32
*E. coli* NCTC 10418	1/1	2/2	1/1	1/1
*E. coli* NCTC 13846	2/2	4/8	2/2	1/1
*E. coli* ATCC 2340	2/2	4/4	2/2	1/1
*K. pneumoniae* ATCC 43816	2/2	8/8	2/2	1/2
*K. pneumoniae* ATCC 1705	4/4	8/8	2/2	1/1
*K. pneumoniae* ATCC 2342	2/2	4/8	2/2	1/1
*A. baumannii* ATCC 747	2/2	2/4	1/1	1/1
*A. baumannii* ATCC 1710	2/2	4/4	1/1	1/1
*A. baumannii* ATCC 3252	2/2	4/4	2/2	1/1
*E. cloacae* ATCC 2341	4/4	8/8	2/2	1/1
*P. aeruginosa* PAO1	8/8	8/16	8/8	1/1
*P. aeruginosa* ATCC 9027	8/16	16/16	2/2	1/1
*P. aeruginosa* ATCC 2108	16/32	64/64	8/16	2/4

**Table 4:** HC_10_ (µM), IC_50_ (µM), and SI values of A4 and its analogues.

Peptide	HC_10_ ^a^	IC_50_	GM _HC10&IC50_ ^b^	GM _MIC_			SI ^c^		
				Gram-Positive	Gram-Negative	All	Gram-Positive	Gram-Negative	All
A4	39.1	19	27.3	2.6	3	2.8	10.5	9.1	9.8
A4-1	>128	45.9	108.4	13.9	6.1	9.2	7.8	17.8	11.8
A4-2	128	140.9	134.3	12.1	2.1	5	11.1	64.0	26.9
A4-3	76.9	68.1	72.4	4	1.1	2.1	18.1	65.8	34.5

^a^ HC_10_, peptide concentration causing 10% haemolysis of horse erythrocytes. ^b^ GM, geometric mean. The geometric mean HC_10_/IC_50_ was calculated from the HC_10_ and IC_50_ values. For peptides that showed no detectable haemolysis at 128 μM, an HC_10_ value of 256 μM was assigned for calculation purposes. ^c^ SI, selectivity index. SI was calculated as the ratio of GM _HC10/IC50_ to GM _MIC_.

## Data Availability

The original contributions presented in this study are included in the article. Further inquiries can be directed to the corresponding authors.
